# Superficial basal cell carcinoma treated with curettage followed by topical imiquimod cream: A retrospective study

**DOI:** 10.1016/j.jdin.2023.08.017

**Published:** 2023-09-09

**Authors:** Andrew L. Ondo, James D. Kerner, Isaac P. Ondo, Jacob V. Kerner, Hossein Mousavi, Patrick Trainor, Stuart D. Shanler

**Affiliations:** a Department of Dermatology, University of New Mexico School of Medicine, Albuquerque, New Mexico; b Epiphany Dermatology, Las Cruces, New Mexico; c University of Rochester, Rochester, New York; d Department of Molecular, Cellular and Developmental Biology, University of California Santa Barbara, Santa Barbara, California; e Department of Chemistry and Biochemistry, New Mexico State University, Las Cruces, New Mexico

**Keywords:** administration, basal cell carcinoma, cancer, curettage, cutaneous drug, imiquimod, immunomodulation, medical oncology, treatment related

*To the Editor:* Basal cell carcinoma (BCC) is the most common skin cancer worldwide; up to 30% are the superficial subtype (sBCC), representing a substantial tumor burden.^1^ It has been reported that curettage followed by daily topical application of imiquimod 5% cream for 6 weeks for a group of patients with either superficial or nodular BCC had no observed recurrences, though follow up was limited to only 12 months.^2^^,^^3^ We have found this treatment effective, and desired to determine the long-term recurrence rates for the sBCC subtype in a group of patients large enough to stratify outcomes by tumor location and average length of treatment required to reach a typical clinical response to imiquimod cream in this setting.

This retrospective evaluation of patients with sBCC treated by curettage followed by topical imiquimod 5% cream (C&I), included all immunocompetent patients with a primary sBCC treated in our practice between January 2008 and December 2012. A control group consists of all patients treated for sBCC by any other method during the same period. Maximum follow up was 132 months.

The C&I treatment method was the same as previously published for cutaneous squamous cell carcinoma in situ.^4^ Briefly, this included curettage of the tumor and 2-4 mm of peritumoral skin followed 2 days later (as it was well tolerated, compared with 7 days originally reported) by daily application of imiquimod 5% cream to the lesion and about ½ inch around until a typical response to imiquimod cream (erythema, crusting, superficial erosion) was observed. If no inflammation was observable at 2-weeks, patients increased to twice daily. Few patients did not have a typical response to imiquimod (only 13/623 patients required more than 4 weeks of daily imiquimod therapy).

Patient characteristics are presented in (Table I). In the treatment group, the average number of days of imiquimod application was 19 (5-41). Local reactions to imiquimod (itching, soreness and stinging) occurred in 62% (388/623) of patients but were not severe enough to discontinue treatment. Systemic symptoms (fatigue, malaise, or nausea) occurred in 2.1% (13/623) of patients; treatment was discontinued and symptoms resolved in all patients in less than 1 week.

**Table I:** Patient demographics and results

Patient characteristics	Treated C&I	Control	SMD
Total no. patients	623	72	
Male (%)	375 (60)	46 (64)	0.082
Female (%)	248 (40)	26 (36)	0.082
Age, mean (range), y	68.6 (28-99)	69 (39-88)	0.043
Patient follow up, median (range), m	87 (0-132)	98 (0-132)	0.073
Total no. tumors	926	121	
Tumor diameter, mean (range), mm	9.2 (4-43)	8.7 (3-24)	0.113
Results			
Recurrences, no.	19	18	
KM 10 year survival, % (95% CI)	97.5 (96.4, 98)	84.1 (77.2, 91)	<0.0001
Control group by treatment type: recurrences/total (% recurrence)			
Destruction alone		8/75 (10.6)	
Imiquimod 5% cream alone		4/20 (20)	
5-Fluorouracil 5% cream alone		5/17 (29)	
Excision		1/7 (14)	
Combination imiquimod 5%/5-fluorouracil 5% creams		0/2 (0)	

The log rank test was used to compare the KM 10 year survival rate.

*C&I*, Curettage followed by daily imiquimod 5% cream; *CI*, confidence interval; *KM*, Kaplan-Meier; *SMD*, standardized mean difference.

The overall Kaplan–Meier (KM) 10 year tumor-free survival was 97.5% with control group 84.1%, control group details in (Table I). In the C&I group, facial lesions alone were 94.3%, trunk and extremities 99% to 100%, with individual locations detailed, (Table II). During the follow up period 457 patients in the C&I group developed a new nonmelanoma skin cancer and 94% (429/457) chose this treatment method for at least 1 additional skin cancer.

**Table II:** Summary locations for cutaneous superficial basal cell carcinoma treated by curettage followed by imiquimod 5% cream

	Location	No.	No. (%) recurrence	Kaplan–Meier estimated 10-year recurrence-free % survival (95% CI)	Diameter range	Average diameter (mm)
Face						
	Forehead	33	0 (0%)	100.0 (100.0, 100.0)	4-30 mm	7.6
	Temple	23	0 (0%)	100.0 (100.0, 100.0)	4-15 mm	7.4
	Cheek	95	3 (3.2%)	95.3 (90.2, 100.0)	4-30 mm	7.7
	Nose	52	6 (11.5%)	84.4 (73.5, 97.0)	4-28 mm	6.3
	Ear	5	0 (0%)	100.0 (100.0, 100.0)	5-12 mm	7.4
	Cutaneous Lip	2	0 (0%)	100.0 (100.0, 100.0)	4-8 mm	6.0
Non-face						
	Scalp	18	2 (11.1%)	86.9 (71.4, 100.0)	6-22 mm	10.3
	Neck	82	5 (6.1%)	92.5 (86.3, 99.1)	4-29 mm	10.9
	Arm	123	1 (0.8%)	99.1 (97.3, 100.0)	4-40 mm	9.1
	Chest	110	1 (0.9%)	98.8 (96.6, 100.0)	4-25 mm	9.5
	Back	252	1 (0.4%)	99.6 (98.7, 100.0)	4-32 mm	9.4
	Abdomen	18	0 (0%)	100.0 (100.0, 100.0)	7-43 mm	12.5
	Leg	113	0 (0%)	100.0 (100.0, 100.0)	4-32 mm	10.4
	Total	926	19 (2.1%)	97.5 (96.4, 98.6)	4-43 mm	9.2

*CI*, Confidence interval.

Treating cutaneous sBCC by C&I was tolerated well with an average of only 19 days of imiquimod application required to reach an appropriate response, with a high overall KM recurrence-free survival (97.5%) at 10 years, significantly higher than the control group (84.1%), with average follow up greater than 6 years (80 months). Notably lower recurrence-free survival rates were seen on the nose (84.4%) and scalp (86.9%). Limitations include the retrospective single-center design; prospective studies are warranted.

## Conflicts of interest

None disclosed.
